# Hormesis as a Particular Type of Plant Stress Response

**DOI:** 10.3390/plants14243815

**Published:** 2025-12-15

**Authors:** Agnieszka Siemieniuk, Małgorzata Rudnicka, Gabriela Jemioła, Eugeniusz Małkowski

**Affiliations:** Plant Ecophysiology Team, Institute of Biology, Biotechnology and Environmental Protection, Faculty of Natural Sciences, University of Silesia in Katowice, 40-007 Katowice, Poland; agnieszka.siemieniuk@us.edu.pl (A.S.); gabriela.jemiola@us.edu.pl (G.J.); eugeniusz.malkowski@us.edu.pl (E.M.)

**Keywords:** hormesis, plant stress, toxic factor, acclimation

## Abstract

Plants are continuously exposed to various abiotic and biotic stress factors, which influence their growth, productivity, and ecological fitness. This paper clarifies the concept of hormesis as a distinct low-dose stress response to toxic substances and presents its relationships with other plant stress phenomena. Based on evidence from the published literature, hormesis can be considered a particular type of acclimation because it involves temporary, non-heritable physiological adjustments to mild toxic stress. It is induced by low doses of toxic substances (e.g., cadmium (Cd), lead (Pb), and chromium (Cr)) and characterised by stimulated growth resulting from the moderate activation of defence mechanisms, including antioxidant activity, reactive oxygen species regulation and/or enhanced photosynthetic efficiency, as well as increased auxin content. We propose that the fundamental parameter for identifying hormetic responses should be plant growth, expressed as shoot biomass or elongation, as analyses of single physiological traits alone are insufficient. Furthermore, growth stimulation caused by factors with physiological functions (physiological factors) such as light, temperature or mineral nutrients should be regarded as forms of acclimation rather than hormesis. These assumptions provide a clearer framework for future studies on plant stress physiology.

## 1. Introduction

In natural conditions, plants are constantly exposed to a variety of stress factors. The resulting stress, as a state of deviation from homeostasis, represents the crucial factor of selective pressure and underlies such important plant processes as growth, yield, reproduction or the occupation of environmental niches. The phenomenon of plant stress is also associated with processes such as adaptation, acclimation and hormesis [1].

Hormesis is a phenomenon that has been known and studied in humans and animals for many years [2,3,4,5,6,7,8,9]. Studies in which hormesis has been observed in plants have also been conducted for many years. However, this specific plant response to any toxic agent initially did not attract widespread interest among plant biologists [10]. Over the past 25 years, interest in hormesis in plants has increased significantly. Many new experimental studies have been published, with authors describing hormesis induced by different toxic agents in many plant species [11,12,13,14,15,16,17,18,19,20,21]. A significant review paper that tried to describe the terminology and precisely define hormesis in plants was the article by Poschenrieder et al. [10]. They recommended that the term hormesis can be used for the stimulation of plant growth in response to treatment with low concentrations of toxic metals in cases where the underlying mechanisms remain to be elucidated. If the mechanism responsible for growth stimulation is identified, such terms as amelioration, defence gene activation, priming or acclimation should be used. More recently, the term priming has been assigned to biotic factors, which are not the subject of this review [22]. Although Poschenrieder et al. [10] demonstrated that the phenomenon of hormesis could be classified as one of the well-known processes (acclimation, amelioration), these terms are not used in papers investigating hormesis, e.g., [14,16,19,21], even if a hormetic stimulation mechanism is proposed. In our opinion, this classification is partially true; however, the mechanism of hormesis may constitute a distinct type of one of the aforementioned processes. In addition, it seems that the terms “adaptation”, “adaptive mechanism” or “adaptive traits” in papers on hormesis are used too frequently or attributed to an inappropriate meaning [14,23,24].

In recent years, several new review papers on hormesis have been published [8,23,25,26,27,28,29,30,31,32,33]. In some of these papers, hormesis is defined as the stimulating action of any factor, including factors with physiological functions, such as light, water, CO_2_, or mineral nutrients [28,33,34]. This approach does not seem to be fully justified. For many years, the plant response to changes in the intensity or concentration of factors with physiological functions has been called acclimation [35,36,37,38,39]. Furthermore, Poschenrieder et al. [10] showed that the concentration–growth response curve for mineral nutrients (e.g., N, Fe, Zn) has an entirely different course than for toxic trace elements (e.g., Cd, Cr, Pb). This indicates that the linking of factors with physiological functions to hormesis is based on erroneous assumptions. As a result, the aim of our review article is to highlight the erroneous assumptions about the phenomenon of hormesis that have recently appeared in the literature, as well as to present the relationships among hormesis, acclimation, and adaptation. We hope that our article will open up a wide scientific discussion on the phenomenon of hormesis. The reviewed literature was collected from the scientific databases, covering publications from 2000–2025.

## 2. Plant Responses to Stress Factors

**Figure 1:**
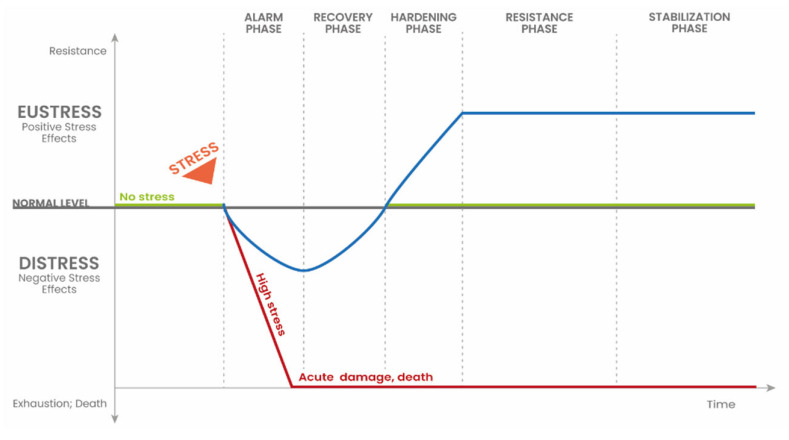
Stress response phases (on the basis of [40]; modified). The green line illustrates the behaviour of the plant in the absence of the stress factor. The blue line illustrates the effect of a stress factor at low intensity, causing eustress. Eustress is associated with an increase in the resistance of the plant to stressors. The red line illustrates the action of the stress factor at a high intensity, causing the occurrence of distress. Distress results in damage to the plant or death.

Physiological factors (factors with physiological functions), in their optimal range, are essential for plant metabolism and developmental processes. However, the occurrence of these factors outside the physiological range contributes to a phenomenon known as stress [40]. Stress factors can be divided into abiotic (i.e., drought, flooding, fluctuations in oxygen and carbon dioxide levels, excess light or inadequate temperature) and biotic (i.e., microorganisms), or in terms of the influence they have on the plant: positive effects (eustress) and adverse effects that may even lead to the death of the plant (distress) [40,41,42]. The relationships between eustress and distress are shown in Figure 1.

Four main phases of the stress response can be distinguished: the alarm phase, the recovery phase, the hardening phase, and the stabilisation phase (Figure 1) [40,41,43]. In the alarm phase, a series of events occurs at the molecular level, e.g., signal perception and transduction or the activation of transcription factors. In the recovery and hardening phases, the plant responds to the stress factors through repair mechanisms (i.e., changes in gene expression or the proteome). During the stabilisation phase, all changes at the molecular level result in the establishment of a new physiological state optimal for the stress conditions. If the plant is under hard/high stress (distress), it enters an exhaustion phase, leading to severe damage and, ultimately, death [40,41,42,43,44,45]. However, a lower-intensity stressor may not be destructive, but may instead trigger plant tolerance to stress. Sometimes, it can even lead to the phenomenon of temporary enhanced plant growth and productivity, known as hormesis.

## 3. The Phenomenon of Hormesis

**Figure 2:**
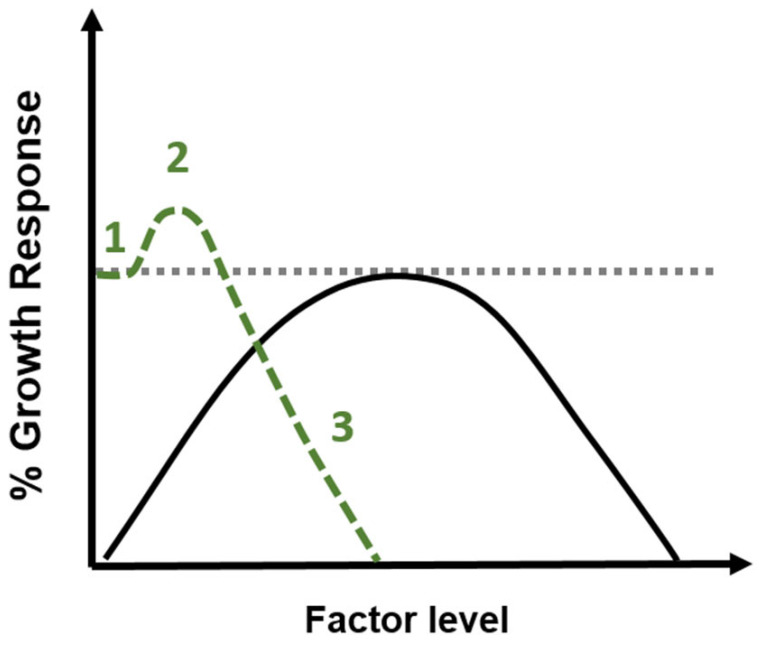
Model of plant growth response to various factors. The black solid line shows the reaction of the plant to physiological factors such as macro- and micronutrients, light or water. The shape of the response curve to physiological factors indicates a reduction in growth when a deficiency or an excess of essential factor occurs. However, when the levels of physiological factors are within the optimal range, a maximum growth response (100%) is recorded. The green dashed line shows a typical hormesis curve of the response to toxic factors. The inverted “J” shape is characteristic and can be divided into three phases: 1—when no changes in growth response are observed with increasing dose; 2—when beneficial effects of stress factors are observed; and 3—when the growth response of plant is inhibited. The dotted line symbolises the maximum growth response (100%) when optimal growth conditions occur, and there is no interaction with the stress factor.

Hormesis was defined many years ago as a beneficial response, in plants, to a low dose of a toxic substance [10,46]. In recent years, this concept has been expanded to include the action of other factors, not just toxic ones. Currently, hormesis is defined as the action of all factors, including physiological ones, such as light, water, or macro- or micronutrients ([47,48,49] and literature herein). Meanwhile, the model of response describing a plant’s reaction to physiological factors differs significantly from the typical hormesis curve of response (Figure 2). A comparison of the two curves clearly shows that the plant response to a physiological factor is different from the hormetic response to a toxic factor. Thus, the positive response of plants to physiological factors should not be considered a hormesis phenomenon. Moreover, numerous authors define hormesis by limiting it to only two phases of this process, which depend on the intensity of the stress factor: low-dose stimulation and high-dose inhibition [31,32,50]; on the other hand, the common and widely accepted scientific model describing the phases of hormesis takes into account the phase of the sub-hormetic level or even the absence of the stress factor [30,51]. This pattern only fits stressors originally considered hormetic, such as non-essential ones (Figure 2).

As mentioned above, the primary assumption of the hormesis model is that responses are amplified/stimulated (i.e., upregulated) to allow adjustment to low doses of a toxic factor. For example, this could involve increasing biological resistance, in contrast to the detrimental, irreversible effects of high doses [52,53]. In accordance with recent hypotheses, hormesis is the consequence of moderate defence activation, which results in the elimination of damage and the enhancement of photosynthesis and dark respiration efficiency. Consequently, the assimilation of energy by the plant exceeds its dissimilation (leading to a positive energy budget), which promotes plant growth and productivity [32]. Nevertheless, it should be stressed that photosynthesis stimulation is not always necessary for hormetic growth stimulation, as demonstrated by Małkowski et al. [16] in maize treated with low Cd concentrations.

However, reviewing the scientific literature, it can be concluded that hormesis no longer has its original meaning of a dose–response relationship and now comprises a wide range of different concepts and mechanisms, such as amelioration, acclimation, eliciting stress responses, crosstalk in stress signalling, and epigenetic effects [10]. Firstly, it should be emphasised that the hormesis phenomenon should be defined as the temporary beneficial effect of mild stress (eustress) caused by toxic substances on the growth response and fitness of plants [10,40]. Additionally, when analysing the beneficial effects of low concentrations of a stress factor on plants, a thorough assessment of the probable mechanism of action of a given stressor is necessary. The hormetic action of a stress factor should include direct and beneficial interactions and indirect actions, or both of them, through the induction of defence mechanisms. Defence mechanisms include signal perception, signal transduction and the activation of defence mechanisms at the transcriptional and/or post-transcriptional level, leading to increased stress resistance or tolerance [10,54,55,56]. Among these mechanisms are primarily the production of reactive oxygen species (ROS), an increase in antioxidant enzyme activity, changes in nutrient uptake and translocation and associated changes in enzyme activity. In addition, these mechanisms should include increases in photosynthetic pigments, changes in the expression levels of specific genes, modifications to metabolic pathways and increases in the efficiency of the photosynthetic process [10,23,27,57]. On the other hand, all mechanisms that do not occur in planta, such as the interaction between the stress factor and metal ion out of the plant organism (known as the ion amelioration phenomenon), must be excluded. When investigating the hormesis phenomenon, the aspect of time should also not be excluded, as the hormetic response observed after a few minutes of exposure may differ markedly from that occurring after hours, days or even weeks [58]. Observing several time points is worthwhile to understand the dynamics of the changes accompanying hormesis [10,59,60].

Defence mechanisms induced in the hormetic response include the alleviation of oxidative stress by increasing the activity of antioxidant enzymes such as superoxide dismutase, ascorbate peroxidase, and catalase [12,61,62] and increasing the production of glutathione, metallothioneins and phytochelatins (PCs) [63]. In addition, low concentrations of stress hormones such as ABA accompanying this phenomenon promote an increase in photosynthetic pigments (chlorophyll a, chlorophyll b and carotenoids) and photosynthetic efficiency [64,65,66,67,68].

**Figure 3:**
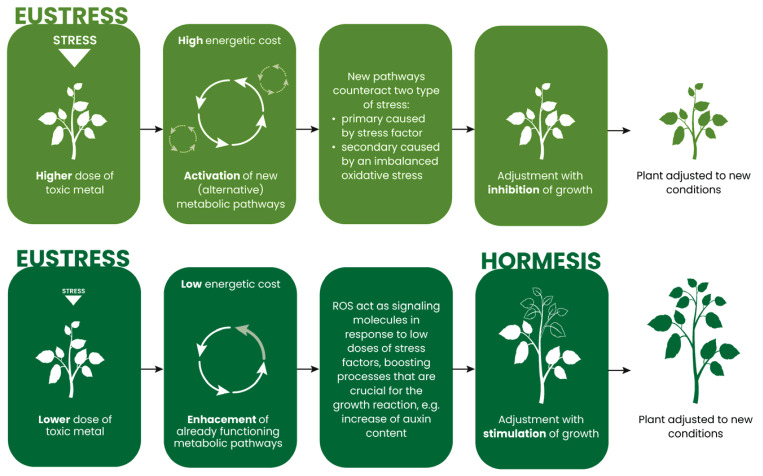
Probable mechanism of plant growth stimulation during hormesis. Upper row: additional metabolic pathways are activated during typical acclimation, with a significant metabolic cost due to the higher level of oxidative stress, the effects of which must be alleviated through new pathways (dark respiration, secondary metabolites synthesis, and other defence processes), resulting in restricted plant growth. Lower row: during acclimation with hormesis, when oxidative stress only reaches the signalling level, the already functioning metabolic pathways are enhanced. The energy cost is low, and the increased metabolic activity and auxin content stimulate growth. The hypothetic hormesis-related processes are as follows: IAA biosynthesis and signalling, sugar metabolic pathways, and photosynthesis-related processes.

To date, we do not know the exact mechanism determining the stimulation of plant growth during hormesis. We suggest that this is probably related to the energy costs of the plant. In the case of typical acclimation, in order to adjust to the presence of a stress factor in the environment, additional metabolic pathways which are distinct from the existing metabolic processes are activated, with a significant energy cost associated with this. In order to reduce energy costs, the plant first inhibits growth (Figure 3, top row). In contrast, during hormesis, only the already functioning metabolic pathways are stimulated. As a result, energy costs are low and the enhanced metabolism enables the plant to increase biomass despite the stressor (Figure 3, bottom row). Nevertheless, the hormetic responses described in the literature, i.e., the direct and indirect reactions and the metabolic pathways activated, correspond to processes characteristic of the classical response to a stress factor, i.e., acclimation. Therefore, the present study proposes to classify hormesis as a particular type of this typical plant adjustment process.

## 4. Other Stress-Related Processes

Variable environmental conditions and stress factors can induce transient individual responses or fixed changes in the plant genotype (Table 1). Adaptive changes are heritable and irreversible and underpin the natural evolution of organisms according to the principle that the better-adapted organism wins (‘survival of the fittest’ notion) [69]. While genotype changes resulting from mutation or gene transfer are relatively rapid, their fixation through natural selection and maintenance in the population is slow and lengthy [70].

Adaptations, as permanent changes in the plant genotype, should be distinguished from changes in the life of individuals in response to a new unfavourable changes in the environment caused by a stress factor. Such changes, part of a process called acclimation, are generally transient, non-heritable, and mainly disappear when the stress factor no longer exists. Although epigenetic mechanisms typical for acclimation (DNA and histone methylation or acetylation) can extend the duration of acclimation and make it heritable by mitotic and meiotic divisions [71], this is a relatively rare phenomenon. Much more common in plants is the resetting of epigenetic mechanisms and a return to the state prior to the action of the stressor, with an evolutionary advantage for plants living often under highly variable environmental conditions [72].

Acclimation mechanisms are activated in plants primarily in response to an excess or deficiency of a factor necessary for the plant to function correctly. Factors activating the acclimation response are, for example, elevated CO_2_ [35], reduced oxygen levels, flooding [73,74], drought [75], high or low temperatures [76,77], and excess or insufficient light [78,79]. Also widely reported by many authors is acclimation in response to macro- and micronutrient imbalances [80,81,82,83,84]. Acclimation has also been described as a response to toxic nonessential factors such as heavy metals (Cd, Pb) [85,86,87].

Based on the literature reviewed, it is clear that the hormesis phenomenon described by many authors so far relates to the life of the individual, and the hormetic response they observed was certainly not related to sustained changes at the genome level [14,15,16,17,18,19,20]. It can thus be concluded that the hormesis phenomenon cannot be linked with adaptation mechanisms. Consequently, the terms ‘adaptation’ and ‘adaptive traits’ should not be used to describe this process.

**Figure 4:**
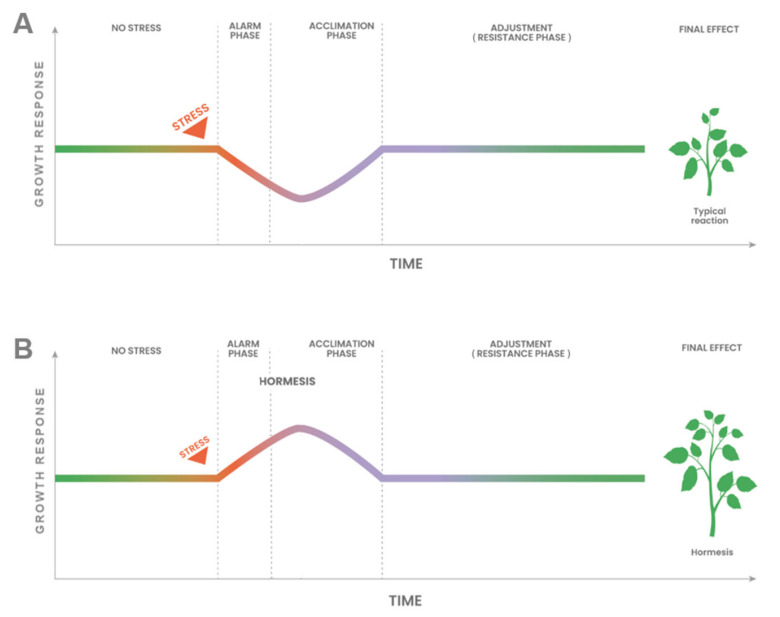
Comparison of the typical acclimation response (**A**) with a particular type of acclimation, which is hormesis (**B**).

Hence, if hormesis is not an adaptive process, it raises the question of whether it is somehow related to the process of acclimation. The preceding discussion on stress and acclimation indicates that analogous processes are triggered in plants during both acclimation and hormesis [26,27,28,88]. In accordance with the foregoing, the hormesis phenomenon should be regarded as a distinct form of acclimation. Figure 4A shows a typical growth response during the acclimation of a plant to stress conditions (eustress).

Perception of a stress stimulus (alarm phase) at a low or moderate dose usually decreases the plant growth response. Then, as a result of the activation of repair processes during acclimation, the growth returns to the initial rate (Figure 4A, resistance phase) when the plant has adjusted to the stress conditions. Ultimately, the plant produces a smaller biomass than the control plants. In the case of hormesis (very low or low doses of the toxic factor), presented in Figure 4B, no significant decrease in growth response is observed during the alarm phase. Moreover, the growth rate increases during the acclimation phase compared to the growth response before the stressor is applied. Once acclimation is completed and the plant has adjusted to the new environmental conditions, the growth response decreases to the optimum rate for the plant, probably the rate observed in control plants. The effect of faster plant growth during acclimation is ultimately increased biomass relative to that of control plants.

Accepting that the hormesis phenomenon is an example of acclimation has many implications. Since acclimation is related to the adjustment of plants to changed environmental conditions [89], only the terms “adjustment”, “adjustment traits”, etc., should be used in relation to hormesis. By contrast, such terms as “adaptation”, “adaptation mechanisms”, and “adaptation traits” should not be used. Acclimation is the response of a plant to changes in the environment. Therefore, the response of plants to changes in physiological factors, such as temperature, light, soil moisture, and macro- and micronutrients, should be considered a typical acclimation response and not hormesis, even when growth stimulation is observed. In contrast, growth stimulation under the influence of low doses of toxic substances, for example cadmium (Cd), chromium (Cr), mercury (Hg) or lead (Pb), should be considered hormesis. This assumption of hormesis is also proposed by other authors [10,29,90]. In view of the aforementioned points, environmental hormesis also should be re-defined and re-described, as the study revealed that this topic often includes considerations of physiological factors such as light, temperature, soil moisture, or nitrogen [24,49].

## 5. Experimental Assessment of Hormesis

Currently, a holistic approach to various issues in plant biology is becoming increasingly important [91,92,93]. In our opinion, such a holistic approach should also apply to research on hormesis in plants. Therefore, we propose that the parameter determining the occurrence of hormesis should be the growth of whole plants or shoots, determined by elongation growth, or fresh or dry weight. At the same time, measuring root growth alone seems unjustified. Root biomass accounts for 15 to 30% of shoot biomass, so studying roots alone to observe hormesis seems unreliable. For example, Szopiński et al. [94] observed in hydroponic cultures in *Arabidopsis arenosa* a 5.5-fold lower dry biomass of roots compared to shoots. In turn, Rusinowski et al. [95] found in pot studies on maize that the dry weight of shoots was 3.0 to 4.8 times greater than that of roots, depending on the soil type. Moreover, shoot biomass is used to determine the yield of various plant species, mainly grasses but also dicotyledons [96,97,98]. These results confirm that shoot biomass is the most important factor in determining plant growth. Furthermore, examining individual parameters, such as chlorophyll content, or only one process, for example photosynthesis, is not sufficient to conclude that hormesis has occurred. Therefore, the results of studies on hormesis that present findings concerning only roots [11,99] or individual processes, such as photosynthesis [17,29,100], or one parameter, such as chlorophyll content [31], should be regarded with great caution. In the case of the aforementioned studies, it would be valuable to repeat them and supplement them with the growth response of shoots or whole plants in order to be sure that hormesis actually occurred.

If a single physiological process is considered, it is easy to overlook actual hormesis, that is, the stimulation of plant growth. For example, Małkowski et al. [16] showed in maize treated with 10 µM Cd that photosynthetic rate and PSII activity were significantly lower compared to the control, while at the same time, stimulation of the elongation growth of Cd-treated seedlings, i.e., a hormesis phenomenon, was observed. If only PSII activity or photosynthetic rate was studied in this case, it would have been concluded that Cd at this concentration and in this experimental setup did not induce hormesis, which would have been a mistake. The same authors also studied Pb-induced hormesis in the same experimental setup [16]. The strongest stimulation of maize shoot growth occurred in the presence of 5 µM Pb. However, unlike with Cd, hormetic growth stimulation was accompanied by an increased photosynthetic rate and chlorophyll content. These results indicate that, depending on the metal, hormetic growth stimulation may result from changes in different metabolic pathways of the plant. A typical response for both metals during hormesis was an increase in auxin content and flavonols. However, no increase in H_2_O_2_ content was observed, suggesting the absence of oxidative stress [16]. This does not indicate that Cd did not stimulate ROS generation. Indeed, it must be remembered that an increase in ROS formation alone does not cause oxidative stress. As long as the antioxidant system of the plant is able to maintain the ROS content at a safe level for the plant, no oxidative stress occurs despite the increase in ROS production. It is only when the antioxidant system fails to cope with the removal of ROS formed in the plant that the accumulation of ROS and oxidative stress occur [101]. In turn, an increase in auxin levels may be the main cause of the elevated plant growth response during hormesis, since auxin primarily stimulates cell elongation growth, particularly in the above-ground parts of plants. A similar correlation of the hormetic growth response with a higher content of auxin in plant tissues or the activation of auxin synthesis pathways has been reported [102,103].

On the basis of the examples given above, it must be concluded that hormesis in plants must be studied at the organismal level and not at the level of individual organs or tissues; particularly, it should not be studied at the level of individual physiological processes, such as photosynthesis. The primary parameter that must always be measured is plant growth. Growth measurement can consist of measuring the fresh or dry weight or the elongation growth of the shoots. Of course, whole-plant biomass is also a suitable parameter, although in many cases, it is difficult to measure. Since the shoot biomass is generally 2–4 times greater than the root biomass, the biomass of the aboveground parts is the best parameter for the observation of hormesis.

According to the assumptions given above, we cannot talk about hormesis on the grounds of a root-only response. For example, during drought stress, an increase in the ABA concentration in the plant stimulates root growth relative to that of shoots, the growth of which is strongly inhibited [104]. Based on root biomass alone, one could speak of hormesis. At the same time, however, shoot biomass is sometimes significantly reduced, resulting in the lower biomass of the whole plant so that hormesis does not occur in such a case. Also, other authors consider the organismal level to be the basis when studying hormesis (Table 2) and that for plants, growth is the primary and most important parameter [10]. To conclude, stress can modify plant growth by increasing the growth of certain parts in response to specific stresses, but this usually involves the sacrifice of other parts. The overall effect of stress is better reflected by the accumulation of whole-plant biomass [105].

## 6. Conclusions

Hormesis refers to the stimulation of plant growth under the influence of low concentrations or doses of toxic substances. It is proposed that growth stimulation should be the primary parameter without which one should not speak of hormesis. Measurements of other parameters, for example chlorophyll content, oxidative stress, and photosynthetic rate, are intended to help determine the mechanisms of hormetic growth stimulation. However, they cannot be the only parameters based on which hormesis is established. Since toxic substances induce hormesis, it is not correct to speak of hormesis due to physiological factors such as light, temperature, soil moisture, or micro- or macronutrients. It seems that hormesis can be regarded as specific type of acclimation.

There is a need for more experiments on the role of auxins in hormetic growth stimulation.

## Figures and Tables

**Table 1:** Concise definitions of processes highlight the fundamental differences between them.

Process	Definition
Acclimation	A facultative physiological modification during the individual life of a plant in response to changes in environmental factor(s) necessary for the plant to function correctly, e.g., fluctuations in water, light, macro- and micronutrients, and concentrations of CO_2_ and O_2_, as well as toxic factors as toxic trace elements or toxic organic substances.
Adaptation	A genetically conserved, heritable modification in the genome of plants to survive and reproduce in a hostile environment. Examples of adaptation include photosynthetic processes in CAM and C4 plants or hyperaccumulation of Zn and Cd in *Arabidopsis halleri*.
Hormesis	A beneficial effect of low doses of toxic factor(s), e.g., Cd, Hg, Pb, ozone, and herbicides, on plant growth response and fitness.
Priming	The capacity of plants to memorise environmental stresses, thereby improving their responses to recurrent stress.

**Table 2:** Examples of plant hormetic response based on whole-plant growth parameters.

No.	Hormesis-Inducing Factor	Plant Species	Parameters Analysed	Findings	Publication Details
1	Cadmium	*Polygonatum sibiricum*	plant biomass, photosynthetic efficiency, and polysaccharide content, as well as CAT, SOD and POD activity and MDA content were measured. Moreover, the polysaccharide contents (PCP1, PCP2 and PCP3) were determined.	A hormetic increase in plant biomass was observed, accompanied by enhanced photosynthetic efficiency, increased antioxidant activity, and a higher polysaccharide content.	[19]
2	Glyphosate	*Solanum lycopersicum*	The plant growth reaction (height), photosynthetic pigment content, photosynthetic efficiency, antioxidant enzyme activity (CAT, SOD and POD) and non-photochemical quenching and expression of genes related to NPQ were analysed.	In the case of low doses of glyphosate, an increase in photosynthetic pigment content and photosynthesis efficiency, enhanced antioxidant enzyme activity, and increased plant growth were observed.	[106]
3	Cadmium	*Triticum aestivum*	The plant biomass, root morphology and development, photosynthetic rate, stomatal conductance, intercellular carbon dioxide concentration, and transpiration rate, along with MDA, non-protein thiol (NPT), phytochelatin, and glutathione content, were determined. Moreover, antioxidant enzymes activity was also analysed.	An increase in whole-plant biomass, improved root development and enhanced photosynthetic rate were observed in the presence of a low cadmium concentration. This hormetic response is connected with the enhancement of the photosynthetic and antioxidant system.	[107]
4	Cerium oxide nanoparticles	*Allium sativum*	Growth parameters such as the length and fresh and dry mass of roots and shoots, together with the mitotic index, levels of MDA and ROS, photosynthetic pigment content, and soluble sugar and protein content, were measured.	Hormesis was observed in the presence of cerium oxide nanoparticles, with increased growth, decreased levels of ROS and MDA, increased carbohydrate and protein content, increased photosynthetic pigment levels, and a higher mitotic index.	[108]
5	Cadmium	*Lonicera japonica Thunb.*	The net photosynthesis rate, stomatal conductance, intercellular CO_2_ concentration and transpiration rate, as well as photosynthetic pigment contents, photosynthetic efficiency and total plant biomass were measured.	Increased levels of net photosynthesis rate, photosynthetic pigments, enhanced photosynthetic efficiency, and higher total plant biomass were detected in the case of low cadmium concentration.	[109]
6	Acephate	*Solanum lycopersicum* L.	The shoot height, root length, and dry weight (DW) of shoots and roots, as well as chlorophyll a fluorescence, photosynthesis pigment content, and CAT, SOD and POT activity, were measured. Moreover, the expression levels of genes involved in the photosynthesis antenna were also analysed.	An increase in plant biomass and photosynthetic rate, as well as increased photosynthetic pigment content, was observed in the presence of low doses of acephate. A similar effect was recorded for genes participating in photosynthesis.	[110]
7	Cadmium, Chromium, Lead	*Cardamine hirsuta*, *Poa annua*, *Stellaria media*	The root and shoot dry biomass, number of nodes, leaf area, and photosynthetic pigment content were analysed.	In the presence of chromium for all species tested, the dry biomass of both the roots and shoots increased, as did the number of nodes. However, in the presence of cadmium, a similar hormetic reaction to that described above was observed only for *C. hirsuta* and *P. annua.* Furthermore, in the case of the latter species, this reaction was also accompanied by an increase in leaf area and in the content of photosynthetic pigments.	[111]
8	Cadmium	*Brassica chinensis* L.	Phenotyping of the whole plant, leaf cell anatomy, shoot fresh biomass, and CAT, SOD and POD activity, as well as H_2_O_2_, O_2_˙, and MDA content, were measured. Moreover, analysis of the level of soluble sugars and sequencing of the transcriptome and metabolome were performed.	*Brassica chinensis* showed an increase in shoot biomass and enhanced he antioxidant system when treated with low levels of cadmium. Moreover, enhanced IAA biosynthesis signalling and the plants’ sugar metabolic pathways were observed.	[103]
9	Cadmium	*Brassica oleracea*	The fresh plant biomass, as well as levels of IAA, GSH, GSSG, glucosinolate and MDA, were measured. Additionally, differences in gene expression were analysed in order to identify hormesis-related ones.	Treatment of *Brassica oleracea* with low cadmium doses resulted in an increase in plant biomass, enhanced auxin biosynthesis and an increase in the ratio of reduced to oxidised glutathione. Moreover, up-regulated genes under low Cd concentrations were identified as potentially related to hormesis, such as a transcription factor regulating the Fe deficiency response; an enzyme catalysing the degradation of GSLs; enzymes modulating the structure of the cell wall; and proteins involved in the photosystem II unit.	[102]
10	Glyphosate	*Coffea arabica*	Plant height, leaf number, leaf area, total dry biomass, CO_2_ assimilation, transpiration and stomatal conductance, carboxylation efficiency, intrinsic water use efficiency, rate of electron transport, photosynthetic efficiency, and content of shikimic acid pathway compounds were analysed.	Treatment with low concentrations of glyphosate increased plant biomass, improved photosynthetic efficiency and caused beneficial changes in morphology and biochemistry (shikimic acid pathway compounds).	[112]
11	Glyphosate	*Toona ciliata*	Plant height and stem diameter, chlorophyll a fluorescence, net carbon assimilation rate, stomatal conductance, transpiration rate, internal CO_2_ concentration and ratio of internal to external CO_2_ were measured. Moreover, morphoanatomical characterisation and visible leaf symptom analyses were performed.	*Toone ciliata* exhibited increased plant height and photochemical yield (photosynthetic rate and carboxylation efficiency) in response to lower doses of glyphosate.	[113]
12	Cadmium	*Celosia argentea*, *Celosia cristata*, *Malva crispa* and *Malva rotundifolia*	The shoot length, leaf area, shoot and root dry biomass, Cd bioaccumulation (BCF) and translocation (TF) coefficients, as well as the tolerance index (TI), were measured.	The introduction of a low cadmium concentration resulted in an increase in both shoot and root biomass in the case of *Celosia argentea*, *Celosia cristata*, *Malva crispa* and *Malva rotundifolia*.	[114]

## Data Availability

No new data were created or analyzed in this study.

## References

[1] Mittler R., Zandalinas S.I., Fichman Y., Van Breusegem F. (2022). Reactive Oxygen Species Signalling in Plant Stress Responses. Nat. Rev. Mol. Cell Biol..

[2] Calabrese E.J., Baldwin L.A. (2002). Applications of Hormesis in Toxicology, Risk Assessment and Chemotherapeutics. Trends Pharmacol. Sci..

[3] Calabrese E.J., Baldwin L.A. (2001). Hormesis: U-Shaped Dose Responses and Their Centrality in Toxicology. Trends Pharmacol. Sci..

[4] Calabrese E.J. (2005). Paradigm Lost, Paradigm Found: The Re-Emergence of Hormesis as a Fundamental Dose Response Model in the Toxicological Sciences. Environ. Pollut..

[5] Kefford B.J., Zalizniak L., Warne M.S.J., Nugegoda D. (2008). Is the Integration of Hormesis and Essentiality into Ecotoxicology Now Opening Pandora’s Box?. Environ. Pollut..

[6] Saul N., Pietsch K., Stürzenbaum S.R., Menzel R., Steinberg C.E.W. (2013). Hormesis and Longevity with Tannins: Free of Charge or Cost-Intensive?. Chemosphere.

[7] Seyed Alian R., Dziewięcka M., Kędziorski A., Majchrzycki Ł., Augustyniak M. (2021). Do Nanoparticles Cause Hormesis? Early Physiological Compensatory Response in House Crickets to a Dietary Admixture of GO, Ag, and GOAg Composite. Sci. Total Environ..

[8] Agathokleous E., Calabrese E.J. (2020). A Global Environmental Health Perspective and Optimisation of Stress. Sci. Total Environ..

[9] Agathokleous E., Kitao M., Calabrese E.J. (2018). Human and Veterinary Antibiotics Induce Hormesis in Plants: Scientific and Regulatory Issues and an Environmental Perspective. Environ. Int..

[10] Poschenrieder C., Cabot C., Martos S., Gallego B., Barceló J. (2013). Do Toxic Ions Induce Hormesis in Plants?. Plant Sci..

[11] Migliore L., Cozzolino S., Fiori M. (2000). Phytotoxicity to and Uptake of Flumequine Used in Intensive Aquaculture on the Aquatic Weed, *Lythrum salicaria* L.. Chemosphere.

[12] Jia L., He X., Chen W., Liu Z., Huang Y., Yu S. (2013). Hormesis Phenomena under Cd Stress in a Hyperaccumulator—*Lonicera japonica* Thunb. Ecotoxicology.

[13] Duman F., Ozturk F., Aydin Z. (2010). Biological Responses of Duckweed (*Lemna minor* L.) Exposed to the Inorganic Arsenic Species As(III) and As(V): Effects of Concentration and Duration of Exposure. Ecotoxicology.

[14] González C.I., Maine M.A., Cazenave J., Sanchez G.C., Benavides M.P. (2015). Physiological and Biochemical Responses of *Eichhornia crassipes* Exposed to Cr (III). Environ. Sci. Pollut. Res..

[15] Debeljak M., van Elteren J.T., Špruk A., Izmer A., Vanhaecke F., Vogel-Mikuš K. (2018). The Role of Arbuscular Mycorrhiza in Mercury and Mineral Nutrient Uptake in Maize. Chemosphere.

[16] Małkowski E., Sitko K., Szopí Nski M., Gieró Z., Pogrzeba M., Kalaji H.M., Ziele´znik-Rusinowska P.Z. (2020). Hormesis in Plants: The Role of Oxidative Stress, Auxins and Photosynthesis in Corn Treated with Cd or Pb. Int. J. Mol. Sci. Artic..

[17] Adamakis I.D.S., Sperdouli I., Hanć A., Dobrikova A., Apostolova E., Moustakas M. (2021). Rapid Hormetic Responses of Photosystem II Photochemistry of Clary Sage to Cadmium Exposure. Int. J. Mol. Sci..

[18] Stamelou M.L., Sperdouli I., Pyrri I., Adamakis I.D.S., Moustakas M. (2021). Hormetic Responses of Photosystem II in Tomato to *Botrytis cinerea*. Plants.

[19] Mengdi X., Wenqing C., Haibo D., Xiaoqing W., Li Y., Yuchen K., Hui S., Lei W. (2021). Cadmium-Induced Hormesis Effect in Medicinal Herbs Improves the Efficiency of Safe Utilization for Low Cadmium-Contaminated Farmland Soil. Ecotoxicol. Environ. Saf..

[20] Wang B., Lin I., Yuan X., Zhu Y., Wang Y., Li D., He J., Xiao Y. (2023). Low-Level Cadmium Exposure Induced Hormesis in Peppermint Young Plant by Constantly Activating Antioxidant Activity Based on Physiological and Transcriptomic Analyses. Front. Plant Sci..

[21] Pfleeger T., Blakeley-Smith M., King G., Lee E.H., Plocher M., Olszyk D. (2012). The Effects of Glyphosate and Aminopyralid on a Multi-Species Plant Field Trial. Ecotoxicology.

[22] De Kesel J., Conrath U., Flors V., Luna E., Mageroy M.H., Mauch-Mani B., Pastor V., Pozo M.J., Pieterse C.M.J., Ton J. (2021). The Induced Resistance Lexicon: Do’s and Don’ts. Trends Plant Sci..

[23] Jalal A., de Oliveira Junior J.C., Ribeiro J.S., Fernandes G.C., Mariano G.G., Trindade V.D.R., Reis A.R. (2021). dos Hormesis in Plants: Physiological and Biochemical Responses. Ecotoxicol. Environ. Saf..

[24] Erofeeva E.A. (2022). Environmental Hormesis of Non-Specific and Specific Adaptive Mechanisms in Plants. Sci. Total Environ..

[25] Oliveira H.C., Seabra A.B., Kondak S., Adedokun O.P., Kolbert Z. (2023). Multilevel Approach to Plant–Nanomaterial Relationships: From Cells to Living Ecosystems. J. Exp. Bot..

[26] Muszyńska E., Labudda M. (2019). Dual Role of Metallic Trace Elements in Stress Biology—From Negative to Beneficial Impact on Plants. Int. J. Mol. Sci..

[27] Carvalho M.E.A., Castro P.R.C., Azevedo R.A. (2020). Hormesis in Plants under Cd Exposure: From Toxic to Beneficial Element?. J. Hazard. Mater..

[28] Erofeeva E.A. (2022). Environmental Hormesis: From Cell to Ecosystem. Curr. Opin. Environ. Sci. Health.

[29] Moustakas M., Moustaka J., Sperdouli I. (2022). Hormesis in Photosystem II: A Mechanistic Understanding. Curr. Opin. Toxicol..

[30] Agathokleous E. (2025). Dosing Requirements to Untangle Hormesis in Plant Science. Trends Plant Sci..

[31] Agathokleous E., Feng Z.Z., Peñuelas J. (2020). Chlorophyll Hormesis: Are Chlorophylls Major Components of Stress Biology in Higher Plants?. Sci. Total Environ..

[32] Erofeeva E.A. (2024). Plant Stress Plant Hormesis: The Energy Aspect of Low and High-Dose Stresses. Plant Stress.

[33] Kolbert Z., Szollosi R., Rónavári A., Molnár Á. (2022). Nanoforms of Essential Metals: From Hormetic Phytoeffects to Agricultural Potential. J. Exp. Bot..

[34] Agathokleous E., Kitao M., Calabrese E.J. (2019). Hormesis: A Compelling Platform for Sophisticated Plant Science. Trends Plant Sci..

[35] Sims D.A., Cheng W., Luo Y., Seemann J.R. (1999). Photosynthetic Acclimation to Elevated CO_2_ in a Sunflower Canopy. J. Exp. Bot..

[36] Luo J., Li H., Liu T., Polle A., Peng C., Luo Z. (2013). Bin Nitrogen Metabolism of Two Contrasting Poplar Species during Acclimation to Limiting Nitrogen Availability. J. Exp. Bot..

[37] Wentworth M., Murchie E.H., Gray J.E., Villegas D., Pastenes C., Pinto M., Horton P. (2006). Differential Adaptation of Two Varieties of Common Bean to Abiotic Stress II. Acclimation of Photosynthesis. J. Exp. Bot..

[38] You L., Zhang J., Li L., Xiao C., Feng X., Chen S., Guo L., Hu H. (2020). Involvement of Abscisic Acid, ABI5, and PPC2 in Plant Acclimation to Low CO_2_. J. Exp. Bot..

[39] Bauerle W.L., Bowden J.D., Wang G.G. (2007). The Influence of Temperature on Within-Canopy Acclimation and Variation in Leaf Photosynthesis: Spatial Acclimation to Microclimate Gradients among Climatically Divergent *Acer rubrum* L. Genotypes. J. Exp. Bot..

[40] Galviz Y., Souza G.M., Lüttge U. (2022). The Biological Concept of Stress Revisited: Relations of Stress and Memory of Plants as a Matter of Space–Time. Theor. Exp. Plant Physiol..

[41] Kranner I., Minibayeva F.V., Beckett R.P., Seal C.E. (2010). What Is Stress? Concepts, Definitions and Applications in Seed Science. New Phytol..

[42] Mosa K.A., Ismail A., Helmy M. (2017). Introduction to Plant Stresses. Plant Stress Tolerance.

[43] Lichtenthaler H.K. (1998). The Stress Concept in Plants: An Introduction. Ann. N. Y. Acad. Sci..

[44] Reddy A.R., Chaitanya K.V., Vivekanandan M. (2004). Drought-Induced Responses of Photosynthesis and Antioxidant Metabolism in Higher Plants. J. Plant Physiol..

[45] Shao H.B., Guo Q.J., Chu L.Y., Zhao X.N., Su Z.L., Hu Y.C., Cheng J.F. (2007). Understanding Molecular Mechanism of Higher Plant Plasticity under Abiotic Stress. Colloids Surf. B Biointerfaces.

[46] Sheppard S.C., Regitnig P.J. (1987). Factors Controlling the Hormesis Response in Irradiated Seed. Health Phys..

[47] Jiao Q., Fan L., Zhang H., Zhang J., Jiang Y., Yang J., Li G., Fahad S., Agathokleous E., Chen Y. (2025). Transcriptomic and Ultrastructural Insights into Zinc-Induced Hormesis in Wheat Seedlings: Glutathione-Mediated Antioxidant Defense in Zinc Toxicity Regulation. Plant Stress.

[48] Erofeeva E.A. (2022). Hormesis in Plants: Its Common Occurrence across Stresses. Curr. Opin. Toxicol..

[49] Erofeeva E.A. (2021). Plant Hormesis and Shelford’s Tolerance Law Curve. J. For. Res..

[50] Agathokleous E., Calabrese E.J., Fotopoulos V. (2024). Low-Dose Stress Promotes Sustainable Food Production. npj Sustain. Agric..

[51] Godínez-Mendoza P.L., Rico-Chávez A.K., Ferrusquía-Jimenez N.I., Carbajal-Valenzuela I.A., Villagómez-Aranda A.L., Torres-Pacheco I., Guevara-González R.G. (2023). Plant Hormesis: Revising of the Concepts of Biostimulation, Elicitation and Their Application in a Sustainable Agricultural Production. Sci. Total Environ..

[52] Kendig E.L., Le H.H., Belcher S.M. (2010). Defining Hormesis: Evaluation of a Complex Concentration Response Phenomenon. Int. J. Toxicol..

[53] Agathokleous E., Belz R.G., Calatayud V., De Marco A., Hoshika Y., Kitao M., Saitanis C.J., Sicard P., Paoletti E., Calabrese E.J. (2019). Predicting the Effect of Ozone on Vegetation via Linear Non-Threshold (LNT), Threshold and Hormetic Dose-Response Models. Sci. Total Environ..

[54] Maksymiec W. (2007). Signaling Responses in Plants to Heavy Metal Stress. Acta Physiol. Plant..

[55] Marino D., Dunand C., Puppo A., Pauly N. (2012). A Burst of Plant NADPH Oxidases. Trends Plant Sci..

[56] Rodrigo-Moreno A., Poschenrieder C., Shabala S. (2013). Transition Metals: A Double Edge Sward in ROS Generation and Signaling. Plant Signal. Behav..

[57] Gururani M.A., Venkatesh J., Tran L.S.P. (2015). Regulation of Photosynthesis during Abiotic Stress-Induced Photoinhibition. Mol. Plant.

[58] Li P., Zhang J., Sun X., Agathokleous E., Zheng G. (2022). Atmospheric Pb Induced Hormesis in the Accumulator Plant *Tillandsia usneoides*. Sci. Total Environ..

[59] Magalhaes J.V., Liu J., Guimarães C.T., Lana U.G.P., Alves V.M.C., Wang Y.-H., Schaffert R.E., Hoekenga O.A., Piñeros M.A., Shaff J.E. (2007). A Gene in the Multidrug and Toxic Compound Extrusion (MATE) Family Confers Aluminum Tolerance in Sorghum. Nat. Genet..

[60] Arroyave C., Barceló J., Poschenrieder C., Tolrà R. (2011). Aluminium-Induced Changes in Root Epidermal Cell Patterning, a Distinctive Feature of Hyperresistance to Al in *Brachiaria decumbens*. J. Inorg. Biochem..

[61] Seth C.S., Kumar Chaturvedi P., Misra V. (2008). The Role of Phytochelatins and Antioxidants in Tolerance to Cd Accumulation in *Brassica juncea* L.. Ecotoxicol. Environ. Saf..

[62] Liu Z., He X., Chen W., Yuan F., Yan K., Tao D. (2009). Accumulation and Tolerance Characteristics of Cadmium in a Potential Hyperaccumulator—*Lonicera japonica* Thunb. J. Hazard. Mater..

[63] Souza L.A., Camargos L.S., Carvalho M.E.A. (2018). Toxic Metal Phytoremediation Using High Biomass Non-Hyperaccumulator Crops: New Possibilities for Bioenergy Resources. Phytoremediation Methods, Management and Assessment.

[64] Kitajima K., Hogan K.P. (2003). Increases of Chlorophyll a/b Ratios during Acclimation of Tropical Woody Seedlings to Nitrogen Limitation and High Light. Plant Cell Environ..

[65] Tang Y.-T., Qiu R.-L., Zeng X.-W., Ying R.-R., Yu F.-M., Zhou X.-Y. (2009). Lead, Zinc, Cadmium Hyperaccumulation and Growth Stimulation in *Arabis paniculata* Franch. Environ. Exp. Bot..

[66] Ying R.R., Qiu R.L., Tang Y.T., Hu P.J., Qiu H., Chen H.R., Shi T.H., Morel J.L. (2010). Cadmium Tolerance of Carbon Assimilation Enzymes and Chloroplast in Zn/Cd Hyperaccumulator *Picris divaricata*. J. Plant Physiol..

[67] Liu Z., He X., Chen W. (2011). Effects of Cadmium Hyperaccumulation on the Concentrations of Four Trace Elements in *Lonicera japonica* Thunb. Ecotoxicology.

[68] Muszyńska E., Hanus-Fajerska E., Ciarkowska K. (2018). Studies on Lead and Cadmium Toxicity in *Dianthus carthusianorum* Calamine Ecotype Cultivated In Vitro. Plant Biol..

[69] Singh V. (2020). Environmental Plant Physiology: Botanical Strategies for a Climate Smart Planet.

[70] Giordano M. (2013). Homeostasis: An Underestimated Focal Point of Ecology and Evolution. Plant Sci..

[71] Gutzat R., Mittelsten Scheid O. (2012). Epigenetic Responses to Stress: Triple Defense?. Curr. Opin. Plant Biol..

[72] Crisp P.A., Ganguly D., Eichten S.R., Borevitz J.O., Pogson B.J. (2016). Reconsidering Plant Memory: Intersections between Stress Recovery, RNA Turnover, and Epigenetics. Sci. Adv..

[73] Hartman S., Sasidharan R., Voesenek L.A.C.J. (2021). The Role of Ethylene in Metabolic Acclimations to Low Oxygen. New Phytol..

[74] Meng X., Li L., Narsai R., De Clercq I., Whelan J., Berkowitz O. (2020). Mitochondrial Signalling Is Critical for Acclimation and Adaptation to Flooding in *Arabidopsis thaliana*. Plant J..

[75] Harb A., Krishnan A., Ambavaram M.M.R., Pereira A. (2010). Molecular and Physiological Analysis of Drought Stress in Arabidopsis Reveals Early Responses Leading to Acclimation in Plant Growth. Plant Physiol..

[76] Hassan M.A., Xiang C., Farooq M., Muhammad N., Yan Z., Hui X., Yuanyuan K., Bruno A.K., Lele Z., Jincai L. (2021). Cold Stress in Wheat: Plant Acclimation Responses and Management Strategies. Front. Plant Sci..

[77] Charng Y.Y., Mitra S., Yu S.J. (2023). Maintenance of Abiotic Stress Memory in Plants: Lessons Learned from Heat Acclimation. Plant Cell.

[78] Mullineaux C.W., Emlyn-Jones D. (2005). State Transitions: An Example of Acclimation to Low-Light Stress. J. Exp. Bot..

[79] Karpiński S., Szechyńska-Hebda M., Wituszyńska W., Burdiak P. (2013). Light Acclimation, Retrograde Signalling, Cell Death and Immune Defences in Plants. Plant Cell Environ..

[80] Bazihizina N., Taiti C., Marti L., Rodrigo-Moreno A., Spinelli F., Giordano C., Caparrotta S., Gori M., Azzarello E., Mancuso S. (2014). Zn^2+^-Induced Changes at the Root Level Account for the Increased Tolerance of Acclimated Tobacco Plants. J. Exp. Bot..

[81] Schulten A., Bytomski L., Quintana J., Bernal M., Krämer U. (2019). Do Arabidopsis Squamosa Promoter Binding Protein-Like Genes Act Together in Plant Acclimation to Copper or Zinc Deficiency?. Plant Direct.

[82] Lu X., Vitousek P.M., Mao Q., Gilliam F.S., Luo Y., Zhou G., Zou X., Bai E., Scanlon T.M., Hou E. (2018). Plant Acclimation to Long-Term High Nitrogen Deposition in an N-Rich Tropical Forest. Proc. Natl. Acad. Sci. USA.

[83] Li Y., Yang X., Liu H., Wang W., Wang C., Ding G., Xu F., Wang S., Cai H., Hammond J.P. (2022). Local and Systemic Responses Conferring Acclimation of *Brassica napus* Roots to Low Phosphorus Conditions. J. Exp. Bot..

[84] Tang R.J., Yang Y., Yan Y.W., Mao D.D., Yuan H.M., Wang C., Zhao F.G., Luan S. (2022). Two Transporters Mobilize Magnesium from Vacuolar Stores to Enable Plant Acclimation to Magnesium Deficiency. Plant Physiol..

[85] Carvalho M.E.A., Piotto F.A., Gaziola S.A., Jacomino A.P., Jozefczak M., Cuypers A., Azevedo R.A. (2018). New Insights about Cadmium Impacts on Tomato: Plant Acclimation, Nutritional Changes, Fruit Quality and Yield. Food Energy Secur..

[86] Tokarz K.M., Makowski W., Tokarz B., Hanula M., Sitek E., Muszyńska E., Jędrzejczyk R., Banasiuk R., Chajec Ł., Mazur S. (2020). Can Ceylon Leadwort (*Plumbago zeylanica* L.) Acclimate to Lead Toxicity?—Studies of Photosynthetic Apparatus Efficiency. Int. J. Mol. Sci..

[87] Iven V., Vanbuel I., Hendrix S., Cuypers A. (2023). The Glutathione-Dependent Alarm Triggers Signalling Responses Involved in Plant Acclimation to Cadmium. J. Exp. Bot..

[88] Choudhury F.K., Rivero R.M., Blumwald E., Mittler R. (2017). Reactive Oxygen Species, Abiotic Stress and Stress Combination. Plant J..

[89] Wilson R.S., Franklin C.E. (2002). Testing the Beneficial Acclimation Hypothesis. Trends Ecol. Evol..

[90] Zhang J., Tang Z., Agathokleous E., Zheng G., Xu L., Li P. (2023). Hormesis in the Heavy Metal Accumulator Plant *Tillandsia ionantha* under Cd Exposure: Frequency and Function of Different Biomarkers. Sci. Total Environ..

[91] Hall R.D. (2006). Plant Metabolomics: From Holistic Hope, to Hype, to Hot Topic. New Phytol..

[92] Sze H., Palanivelu R., Harper J.F., Johnson M.A. (2021). Holistic Insights from Pollen Omics: Co-Opting Stress-Responsive Genes and ER-Mediated Proteostasis for Male Fertility. Plant Physiol..

[93] Sanclemente M. (2024). A Holistic Evaluation of Nitrogen Responses in Maize. Plant Physiol..

[94] Szopiński M., Sitko K., Rusinowski S., Zieleźnik-Rusinowska P., Corso M., Rostański A., Rojek-Jelonek M., Verbruggen N., Małkowski E. (2020). Different Strategies of Cd Tolerance and Accumulation in *Arabidopsis halleri* and *Arabidopsis arenosa*. Plant Cell Environ..

[95] Rusinowski S., Szada-Borzyszkowska A., Zieleźnik-Rusinowska P., Małkowski E., Krzyżak J., Woźniak G., Sitko K., Szopiński M., McCalmont J.P., Kalaji H.M. (2019). How Autochthonous Microorganisms Influence Physiological Status of *Zea mays* L. Cultivated on Heavy Metal Contaminated Soils?. Environ. Sci. Pollut. Res..

[96] Antonkiewicz J., Kołodziej B., Bielińska E.J. (2016). The Use of Reed Canary Grass and Giant Miscanthus in the Phytoremediation of Municipal Sewage Sludge. Environ. Sci. Pollut. Res..

[97] Antonkiewicz J., Popławska A., Kołodziej B., Ciarkowska K., Gambuś F., Bryk M., Babula J. (2020). Application of Ash and Municipal Sewage Sludge as Macronutrient Sources in Sustainable Plant Biomass Production. J. Environ. Manag..

[98] Antonkiewicz J., Kołodziej B., Bryk M., Kądziołka M., Pełka R., Koliopoulos T. (2025). Sustainable Management of Bottom Ash and Municipal Sewage Sludge as a Source of Micronutrients for Biomass Production. Sustainability.

[99] Guzmán-Báez G.A., Trejo-Téllez L.I., Ramírez-Olvera S.M., Salinas-Ruíz J., Bello-Bello J.J., Alcántar-González G., Hidalgo-Contreras J.V., Gómez-Merino F.C. (2021). Silver Nanoparticles Increase Nitrogen, Phosphorus, and Potassium Concentrations in Leaves and Stimulate Root Length and Number of Roots in Tomato Seedlings in a Hormetic Manner. Dose-Response.

[100] Agathokleous E., Kitao M., Harayama H. (2019). On the Nonmonotonic, Hormetic Photoprotective Response of Plants to Stress. Dose-Response.

[101] Raja V., Majeed U., Kang H., Andrabi K.I., John R. (2017). Abiotic Stress: Interplay between ROS, Hormones and MAPKs. Environ. Exp. Bot..

[102] Ma X., Zhao X., Zhang Q., Zhou Z., Dou Y., Ji W., Li J. (2022). Comparative Transcriptome Analysis of Broccoli Seedlings under Different Cd Exposure Levels Revealed Possible Pathways Involved in Hormesis. Sci. Hortic..

[103] Li R., Qin M., Yan J., Jia T., Sun X., Pan J., Li W., Liu Z., El-Sheikh M.A., Ahmad P. (2025). Hormesis Effect of Cadmium on Pakchoi Growth: Unraveling the ROS-Mediated IAA-Sugar Metabolism from Multi-Omics Perspective. J. Hazard. Mater..

[104] Mittler R., Blumwald E. (2015). The Roles of ROS and ABA in Systemic Acquired Acclimation. Plant Cell.

[105] Zhang H., Zhao Y., Zhu J.-K. (2020). Thriving under Stress: How Plants Balance Growth and the Stress Response. Dev. Cell.

[106] Wang Y., Cui Y., Li J., Xu N., Shi T., Sun Y., Zhang C. (2024). Glyphosate Hormesis Stimulates Tomato (*Solanum lycopersicum* L.) Plant Growth and Enhances Tolerance against Environmental Abiotic Stress by Triggering Nonphotochemical Quenching. Pest Manag. Sci..

[107] Jiao Q., Li G., Li L., Lin D., Xu Z., Fan L., Zhang J., Shen F., Liu S., Seth C.S. (2024). Hormetic Responses to Cadmium Exposure in Wheat Seedlings: Insights into Morphological, Physiological, and Biochemical Adaptations. Environ. Sci. Pollut. Res..

[108] Viswanathan D., Christudoss A.C., Seenivasan R., Mukherjee A. (2024). Decoding Plant Hormesis: Cerium Oxide Nanoparticles and the Role of Soil EPS in the Growth Dynamics of *Allium sativum* L.. ACS Agric. Sci. Technol..

[109] Thunb L. (2015). Hormesis Effects Induced by Cadmium on Growth and Photosynthetic Performance in a Hyperaccumulator, *Lonicera japonica* Thunb. J. Plant Growth Regul..

[110] Xu N., Sun Y., Wang Y., Cui Y., Jiang Y., Zhang C. (2023). Hormesis Effects in Tomato Plant Growth and Photosynthesis Due to Acephate Exposure Based on Physiology and Transcriptomic Analysis. Pest Manag. Sci..

[111] Salinitro M., Mattarello G., Guardigli G., Odajiu M., Tassoni A. (2021). Induction of Hormesis in Plants by Urban Trace Metal Pollution. Sci. Rep..

[112] Costa R.N., Bevilaqua N.d.C., Krenchinski F.H., Giovanelli B.F., Pereira V.G.C., Velini E.D., Carbonari C.A. (2023). Hormetic Effect of Glyphosate on the Morphology, Physiology and Metabolism of Coffee Plants. Plants.

[113] de Faria G.S., Carlos L., Jakelaitis A., de Freitas S.T.F., Vicentini T.A., Silva I.O.F., Vasconcelos Filho S.C., Lourenço L.L., Farnese F.S., Batista M.A. (2023). Hormetic Effect Caused by Sublethal Doses of Glyphosate on *Toona ciliata* M. Roem. Plants.

[114] Wu M., Luo Q., Liu S., Zhao Y., Long Y., Pan Y. (2018). Screening Ornamental Plants to Identify Potential Cd Hyperaccumulators for Bioremediation. Ecotoxicol. Environ. Saf..

